# Low Correlation between Gait and Quality of Life in Advanced Knee Osteoarthritis

**DOI:** 10.3390/jfmk8020077

**Published:** 2023-06-08

**Authors:** Valentín Freijo, Claudia Navarro, Begoña Molina, Jordi Villalba

**Affiliations:** 1 Department of Physical Medicine and Rehabilitation, Parc Taulí Hospital Universitari, Institut d’Investigació i Innovació Parc Taulí I3PT, Universitat Autònoma de Barcelona, Parc Taulí, 1, 08208 Sabadell, Spain; vfreijo@tauli.cat (V.F.); cnavarroo@tauli.cat (C.N.); bmolina@tauli.cat (B.M.); 2 Department of Orthopedic and Trauma Surgery, Parc Taulí Hospital Universitari, Institut d’Investigació i Innovació Parc Taulí I3PT, Universitat Autònoma de Barcelona, Parc Taulí, 1, 08208 Sabadell, Spain

**Keywords:** advanced knee osteoarthritis, gait, quality of life, activities of daily living, KOOS, EQ-5D

## Abstract

Advanced knee osteoarthritis patients’ gait usually undergoes alterations leading to decreased mobility and lower functional performance, which can result in a worsening of their quality of life (QoL). While several authors have reported a moderate correlation between gait parameters and QoL assessed by generic questionnaires, the literature is scarce. This study aimed to explore the relationship between gait and QoL parameters assessed by a generic and a disease-specific questionnaire in patients with advanced knee osteoarthritis. In this single-centre, prospective, observational study, 129 patients with advanced knee osteoarthritis scheduled for elective total knee replacement were selected. The patients’ gait was evaluated by means of a validated wireless device while they walked 30 m at a comfortable speed. Patient function was also analysed using the Knee Society Score (KSS). QoL was measured with the EQ-5D and the Knee Injury and Osteoarthritis Outcome Score (KOOS) questionnaires. Patients showed a mean walking speed of 0.95 ± 0.19 m/s, a mean cadence of 105.6 ± 9.9 steps/min, and a mean stride length of 1.25 ± 0.17 m on both legs. They presented poor knee status (KSS < 60) and poor QoL, with an EQ-5D of 0.44 ± 0.24 and a total KOOS of 29.77 ± 13.99. Positive low correlations (r <0.5, *p* <0.5) were found only between the speed, propulsion and stride length of both legs, and the overall and ADLs subscale scores of the total KOOS questionnaire. In conclusion, several gait parameters have a significant low correlation with the QoL of patients with advanced knee osteoarthritis, as assessed by an osteoarthritis-specific questionnaire.

## 1. Introduction

Knee osteoarthritis is the most common joint disease and one of the leading causes of poor functional performance in the elderly. It is considered one of the major contributors to patient disability worldwide, thus constituting a significant public health issue, and its prevalence is estimated to increase by 40% in 2025, due to the widespread ageing of the global population, the increase in life expectancy, and the prevalent obesity epidemic. The chief symptom of the disease is knee pain, which results from joint degeneration, cartilage loss, subchondral bone damage, and changes in soft tissue. The progression of the disease may lead to irreversible bone destruction and deformity. This usually results in a worsening of symptoms that have a distinctive and direct influence on multiple factors that affect the physical and mental well-being of the individual, such as gait and quality of life (QoL) [1,2,3,4,5,6,7,8,9].

Alterations in gait patterns are common age-related changes that may be present in all elderly individuals, but can be exacerbated in the presence of musculoskeletal disorders. At more advanced stages of knee osteoarthritis, many patients exhibit gait adaptations that involve unconscious changes to avoid bearing weight on the affected joint, thus preventing an increase in knee pain. This often translates to a lower walking speed and stride length while maintaining a relatively stable cadence, which become more evident as the condition worsens, which can lead to decreased mobility and lower functional performance. These gait parameters are considered predictors of the overall health status and life expectancy of patients, and with the development of more reliable, accessible, and user-friendly analytical systems, they can be easily measured during clinical practice [1,2,9,10,11,12,13,14,15,16,17].

Both the symptomatology and the functional limitations in advanced knee osteoarthritis patients can significantly worsen a patient’s QoL, which is defined by the World Health Organisation as the individual perception of one’s life situation in a broad context, taking into account cultural conditions, individuals’ expectations, and social norms. Being a multidimensional and subjective concept, it is generally accepted that QoL should be assessed by considering a patient’s physical, mental, and emotional state, their level of autonomy, and their social relationships among other factors, by the means of validated generic, anatomy-specific, or disease-specific questionnaires [1,2,3,18].

Given that wait times for treatment can be prolonged for various reasons, we believe it is crucial to gain a better understanding of the factors that may have a stronger negative impact on our patients’ daily well-being. Some studies have reported a moderate correlation between the general health of knee osteoarthritis patients, as assessed by the SF-36 Health survey, and gait parameters, such as single-limb support [19] and the intensity of gait cycles [17]. Similarly, advanced-stage patients’ QoL assessed by EQ-5D has been found to have a moderate correlation with walking and dynamic balance tests [20]. Due to these findings and to what we see in our clinical practice, we believe that gait alterations derived from physical impairment have a highly significant impact on our patient’s activities of daily living (ADLs) and, therefore, in their overall QoL. The literature on the topic is rather scarce, and QoL is usually evaluated by generic tools that, although being official and validated, may lack some assessments that are more closely related to the impact of the pathology on daily life, which can be measured by knee osteoarthritis-specific questionnaires.

The purpose of this study was to find out whether there was a correlation between a series of gait and QoL parameters assessed by a generic and an osteoarthritis-specific questionnaire in patients with advanced knee osteoarthritis. The hypothesis of our study was that gait had a positive correlation with QoL.

## 2. Materials and Methods

Study design and subjects. This is a single-centre, prospective, observational study of 129 consecutively selected patients who presented at our hospital between 2020 and 2021. The study was conducted in accordance with the Declaration of Helsinki and approved by the Ethics Committee of Parc Taulí Hospital Universitari, and all patients gave their written informed consent during the routine interview with their doctor, at which they were informed about their treatment options. The inclusion criteria were defined as presenting advanced-stage knee osteoarthritis (Kellgren and Lawrence grade 3 or 4) [21], being scheduled for total knee arthroplasty at our hospital, and being willing to participate in an unassisted gait test. Patients with neurological conditions, unresolved spinal conditions, cognitive impairment, and those scheduled for revision surgery were all excluded from the study.

We collected patients’ demographic data (age, sex, height, weight, and BMI), as well as their comorbidities using the Charlson Comorbidity Index, and the laterality and severity of the pathology using the Charnley classification (A: Unilateral knee arthritis, B1: Unilateral total knee arthroplasty, opposite knee arthritic, B2: Bilateral total knee arthroplasty, C1: total knee replacement, but remote arthritis affecting ambulation, C2: total knee replacement, but medical condition affecting ambulation, C3: unilateral or bilateral total knee arthroplasty or bilateral total hip replacement) [22].

**Figure 1:**
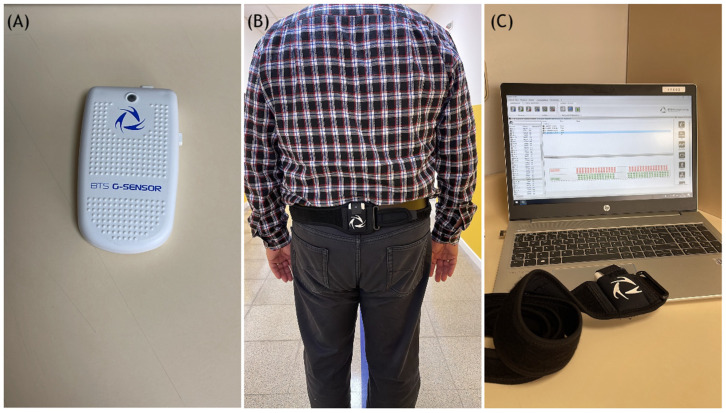
BTS G-Walk system. (**A**) The measuring device is attached to the patient’s waistline via an elastic belt. (**B**) Patient with the device attached by an elastic belt to vertebra S1. (**C**) Collection of data by the BTS G-Walk software.

Gait analysis. Following the protocol of our hospital’s fast-track programme, patients were called in three months before they were due to undergo surgery to inform them about the procedure. During the interview, gait was evaluated by means of the BTS G-Walk gait analysis system (BTS Bioengineering Corp., Quincy, MA, USA). This is a wireless device comprising an inertial sensor with a triaxial accelerometer, a magnetic sensor, and a triaxial gyroscope. Following the manufacturer’s instructions, the triaxial gyroscope was in all cases fastened to the patients’ spine, at the level of the S1 vertebra, by means of an elastic belt. The correct placement of the device was checked by a team of nursing staff, who also ensured that all patients performing the test wore flat closed shoes. Patients were instructed to walk 30 m along a corridor devoid of any obstacles at a leisurely speed (Figure 1). The test lasted approximately 5 min and only required the device to be automatically calibrated. All patients were given explicit instructions on how to perform the test and were allowed to do a few pre-tests to become familiar with the procedure. The device collected data that were subsequently transferred via Bluetooth to a computer. By applying the software provided by the manufacturer, data on speed (m/s), cadence (steps/min), and each leg’s stride length (m) and propulsion (m/s^2^) were obtained (Table 1). BTS G-Walk is a validated instrument for evaluating physical activity. It has been reported to have an inter-instrument correlation coefficient of 0.9–0.99 and an intra-instrument variation coefficient ≤2.5% [23].

Functional evaluation. Knee function was evaluated using the official Spanish version of the Knee Society Score (KSS) (Functional evaluation questionnaire S1). It comprises an Objective Knee score section (with alignment, instability, joint motion, symptoms, patient satisfaction, and patient expectations items) and a Functional score section (with walking and standing, standard activities, advanced activities, and personal activities). Scores range between 0 and 100. A score between 100 and 80 indicates excellent knee function; a score between 79 and 70 indicates good knee function; a score between 69 and 60 indicates fair knee function; and scores below 60 denote poor knee function [24].

Quality of life. To achieve a more comprehensive assessment, patient QoL was evaluated by one generic and one knee osteoarthritis-specific QoL assessment questionnaire, whose items provide complementary information.

The official Spanish version of the self-administered EQ-5D-5L questionnaire was used as the generic QoL assessment tool (Generic QoL evaluation questionnaire S2). EQ-5D-5L includes five dimensions (mobility, self-care, usual activities, pain/discomfort, and anxiety/depression) that can be scored on five levels, ranging from a perfect health state (i.e., no problems at all in a dimension) to the worst possible health state (i.e., problems in a dimension are extreme or disabling). It also includes a visual analogue scale (VAS) to assess the respondents’ health status on the day they were interviewed [25].

In addition, the official Spanish version of the Knee Injury and Osteoarthritis Outcome Score (KOOS) was used as the knee osteoarthritis-specific QoL assessment tool (Knee osteoarthritis-specific QoL evaluation questionnaire S3). This is a self-administered questionnaire designed to assess short- and long-term patient outcomes following knee injury or osteoarthritis. It evaluates pain, symptoms, ADLs, sport and recreation function, and QoL with several items in each category that are scored according to their frequency of occurrence (from never to always), severity (from none to extreme), or certainty (from not at all to totally) [26].

Statistical analysis. The processing of data was carried out using the IBM SPSS Statistics for a Windows software package (IBM Corp., 2017, Version 25.0, Armonk, NY, USA). A descriptive statistical analysis was made of the patients’ clinical and demographic characteristics, their gait scores, and the results of the different questionnaires. The normality of the sample was checked using the Shapiro–Wilk test. The correlation between the gait scores and the results of the functional performance and QoL questionnaires was analysed using the Pearson correlation coefficient. According to the Hinkle et al. range-based classification system (2003) [27], the correlations predicted by the Pearson coefficient are as follows: r <0.29: no correlation; 0.3< r <0.49: low correlation; 0.5< r <0.69: moderate correlation; 0.7< r <0.89: high correlation; and 0.9< r <1: very high correlation. The statistical significance for the analysis of correlations was set at a *p*-value <0.05.

## 3. Results

Of the 129 participating patients, 49 (38%) were male and 80 (62%) were female. The overall mean age was 69.4 years. The rest of the demographic characteristics and those related with the patients’ knee conditions are presented in Table 2.

Table 3 details all the gait scores recorded by the BTS G-Walk gait analysis system. The patients showed a similar stride length and propulsion with both legs.

The results of the function and QoL questionnaires are presented in Table 4. The mean scores on the objective and functional KSS, which were below 60 in all cases, are indicative of poor knee status. Regarding QoL evaluation, patients showed mean poor scores in both the overall EQ-5D (0.44 ± 0.24) and total KOOS (29.77 ± 13.99). The mean scores for all the items of the knee osteoarthritis-specific questionnaire (KOOS) were lower than the self-reported general health status of the generic questionnaire (EQ-5D VAS). The highest impact of the pathology on the KOOS questionnaire was related to the sports/recreation function.

The correlations between the gait scores and the results of the function and QoL questionnaires are shown in Table 5. All correlations were statistically significant, with a *p*-value < 0.05 in all cases. However, the Pearson coefficients were <0.5 in all cases, which indicates a low level, or even an absence, of correlation. A low positive correlation (0.3 < r < 0.49) was only found between speed, stride length (both legs), and right leg propulsion with the overall and ADL subscale scores on the KOOS questionnaire, as well as between left leg propulsion and the KOOS ADL subscale. No correlation (r < 0.29) was found between cadence and the other gait parameters, or between the results of the KSS and EQ-5D questionnaires and the different gait scores.

## 4. Discussion

This study of patients with advanced knee osteoarthritis aimed to analyse the potential relationship between gait, measured by an analytical device while patients performed a simple walking test, and QoL, measured by two validated questionnaires. As there are few studies on this topic, and they mostly reported patients’ QoL using generic questionnaires, we found it important to assess our patients by using both a generic (EQ-5D) and a knee osteoarthritis-specific validated questionnaire (KOOS).

A consensus seems to exist that walking speed ≥ 1 m/s is associated with healthy ageing and a higher survival rate than may be expected when taking only the individual’s age and sex into consideration [28]. Unfortunately, and not surprisingly given their poor medical condition, the patients in this study walked at a slower speed (0.95 m/s) than that achieved by healthy subjects of the same age, which has been found to range between 1.1 and 1.32 m/s by different authors [29,30]. This slower speed is practically identical to that reported in other series of patients with knee osteoarthritis [15,31], and even slightly higher than the 0.85 m/s achieved by the patients treated by Fransen et al. (2022), all of whom were at advanced stages of the disease [32].

With their 105.7 steps/min, our patients exhibited a cadence between 110.9 and 112.8 steps/min, which is lower than what is considered normal for healthy subjects of the same age [29,30]. These values are equivalent or slightly higher than those reported for other patients with knee osteoarthritis (101.3–106.5 steps/min) [15,33]. Moreover, stride length, which was 1.25 cm for both legs, was shorter than the reference values for individuals of the same age, which typically range between 1.33 and 1.47 cm [29]. Nevertheless, the stride length in our patients was longer that that reported in the different articles on patients with knee osteoarthritis that we reviewed, where values ranged between 0.82 and 1.13 cm, depending on the patient’s sex and the extent to which the disease had progressed [15,31,34].

Although we do not know whether there are statistically significant differences between those normative results and our patients’ results, our patients seemed to present gait alterations in line with those reported in the literature, as they decreased their walking speed and shortened their strides to minimise the impact on their affected lower limb.

The poor results obtained in the objective and functional KSS scores, 52.81 and 54.12, respectively, although better than those reported previously for elderly Spanish individuals with knee osteoarthritis (27.4 on the objective KSS and 40.4 on the functional KSS) [35], confirmed a loss of knee function. Consequently, the results of the EQ-5D questionnaire and its VAS scale measuring overall health status, as well as the results of all the KOOS subscales, were indicative of a lower QoL than that enjoyed by healthy individuals of the same age [36,37], and by other series of patients with knee osteoarthritis [38,39]. The functional limitations experienced by patients in advanced stages of knee osteoarthritis can impede the performance of simple tasks such as walking, climbing stairs, squatting, and other movements that compromise the execution of ADLs. Furthermore, symptoms may occur more frequently and even during periods of rest due to severe joint damage, which can alter the patient’s sleep patterns, times, and quality, thereby diminishing their overall QoL [5,8,40].

Regarding the correlations analysed, it is difficult to make comparisons with the findings in the literature given the disparate use made by different authors of the different instruments available to analyse QoL. The strongest correlation observed in the present study was that between speed and the KOOS ADL subscale scores, for which a Pearson coefficient of 0.476 was obtained, indicative of a low positive correlation. This finding, together with the low positive correlation between speed and the overall KOOS score, are in line with the low positive correlation reported by other authors between speed and the general health scores of the SF-36 [41], WOMAC, and SF-12 questionnaires in patients with knee conditions [42]. At the same time, a low positive correlation was found in previous studies between cadence and the scores on the WOMAC and SF-12 questionnaires [42], whereas this study found no correlation whatsoever between cadence and the other QoL parameters. On the other hand, while our patients showed a low positive correlation between the stride length of both legs and the overall and ADL subscale scores of the KOOS questionnaire, previous studies found a complete absence of correlation between cadence and the WOMAC and SF-12 questionnaires [42]. The lack of correlation between the EQ-5D questionnaire and the other parameters studied is in line with the lack of correlation reported for a series of patients with knee osteoarthritis that analysed the same gait parameters as this study [20].

Although it might initially seem reasonable to presume that a decreased QoL is associated with gait impairment due to the biomechanical and functional effects of knee osteoarthritis, the present study found no statistical correlations between QoL and the gait parameters analysed that were strong enough to support such a hypothesis, following our Pearson coefficient categorisation [27]. These results, although not directly comparable with those of other studies given the different methodologies used, seem to be in line with some of the described findings in the literature.

Other authors have previously reported moderate correlations between different gait parameters from those collected in our study and the outcomes of generic QoL questionnaires. However, some of their Pearson correlation coefficients were lower than those obtained in our study. Debi et al. (2011) [19] considered the correlation between SF-36 general health and single-limb support as moderate (r = 0.36), while Chul et al. (2016) [20] seemed to have adopted a similar categorisation by considering that the EQ-5D and the dynamic balance test their patients performed presented a moderate correlation (r = 0.38). Following these examples, at least all our KOOS ADLs correlations with gait parameters, except for cadence, would be considered moderate, being r > 0.38. In the same way, most of the analysed variables would present a low correlation instead of a lack of correlation, which previous studies seem to associate with r = 0. Since there is no official categorisation of correlation based on the Pearson coefficient, we chose a more conservative approach than those of our colleagues, since theirs were unfortunately not properly stated in their articles.

It is extremely complex to determine the exact causes and chronology of the progressive decrease in the QoL of knee osteoarthritis patients. Firstly, the worsening of symptoms—especially pain—is usually accompanied by an increase in compensatory gait actions and a decrease in the patient’s well-being. This often leads to less physical activity due to purely physiological or psychological reasons. Inactivity is estimated to lead to a decline in muscular strength of between 5 and 15%, eventually making a return to physical activity more challenging. All of this can result in a loss of autonomy and personal freedom, affecting both ADLs and social, work, and leisure activities, which in turn further deteriorates the patient’s emotional status [4,8,40,43].

With the aggravation of symptoms, pain may also start to present itself during rest time, once again taking a toll on their QoL. Agata et al. (2022) found that symptoms such as pain, stiffness, and functional disability in patients with knee osteoarthritis had a negative correlation with their QoL. The greater the intensity of the pain they suffered, the lower their QoL and the greater the impact on their perception of well-being, which is associated with mental health and mood. A negative state of mind sustained over time, the feeling of incapacity, and the natural tendency to think about pain may lead to an increased perception of it. This cycle could be broken with surgical treatment, but unfortunately, this may be delayed or not even suitable for some patients [11,40].

Despite not finding a high interconnection between the two of them in our study, other researchers have reported a direct and individual influence of pathology on both QoL and gait. One of the key determining factors in knee impairment appears to be physical activity, as patients with compromised physical activity levels tend to exhibit lower general well-being. Our patients had a compromised physical activity status, as shown in their KSS and Sport/recreation function KOOS scores, due to their restricted functional capacity and avoidance behaviors aimed at managing pain, leading to a more sedentary lifestyle that could ultimately contribute to muscle weakness due to lack of exercise [4,8,40,43].

As mentioned before, the incidence of knee osteoarthritis is likely to escalate in the next few years. Future research should expand the scope of the correlation analysis between gait characteristics and QoL in advanced knee osteoarthritis patients, identifying the key changes in gait patterns as the disease progresses. With the advancement of technologies, such as finite element modelling, it would be interesting to use these digital biomechanical analysis methods to further clarify how the mechanical stress and load changes of an abnormal gait may affect a patient’s pain and, therefore, their QoL. Such research could help identify strategies to mitigate these factors and improve patients’ well-being while they await effective treatments, as well as aiding in the selection of treatment plans [44]. In the field of orthopedics, recent studies have been conducted involving computational simulations of loads on the lower limbs for various purposes, such as defining the risk of ulceration in the diabetic foot based on foot loads during gait [45], optimising footwear to reduce plantar and metatarsal stress when running [46], and analysing mechanical stresses associated with the treatment of knee osteoarthritis patients using proximal fibular osteotomy [47], all of which show promising results.

The chief limitation of this study is related to the fact that gait was analysed in a simple manner as patients walked at a comfortable speed along a 30 m stretch. Although this method allowed an accurate determination of gait scores using the BTS G-Walk system, we did not evaluate gait parameters over long periods of time and while patients were performing their ADLs, such as walking up and down stairs, sitting down, and standing up, and other common tasks that may be particularly challenging for patients with knee osteoarthritis. Moreover, we could not statistically compare our patients’ QoL and gait patterns with a control group, and thus, we could only present a descriptive comparison with previous results reported in the literature. Overall, there is still much to be learned about the relationship between gait patterns and QoL in knee osteoarthritis patients, and continued research has the potential to greatly improve patient outcomes.

This study has demonstrated a significant low positive correlation between several gait parameters that have a significant low correlation with the QoL of patients with advanced knee osteoarthritis, as assessed by an osteoarthritis-specific questionnaire.

## Figures and Tables

**Table 1:** Gait spatiotemporal parameters collected by BTS G-Walk.

Parameter	Definition	Units
Walking speed	Spatial rate at which a person walks, i.e., the distance covered by a person per unit of time.	m/s
Cadence	Number of steps taken per unit of time, using a spontaneous and comfortable walking speed.	steps/min
Stride length	The distance covered during a complete cycle of right or left gait, at a spontaneous and comfortable walking speed.	m
Propulsion	The force generated by the body during walking to propel the body forward, which provides information on the acceleration of the patient’s centre of mass forward during the left and right single support phase.	m/s^2^

**Table 2:** Patient demographic characteristics and classification of the patients’ conditions. Results are presented as mean ± SD or n (%). BMI: Body Mass Index.

	N = 129	Range
Age (years)	69.4 ± 7.9	50–87
Sex		
Males	49 (38%)	
Females	80 (62%)	
Height (m)	1.58 ± 0.09	1.41–1.81
Weight (kg)	84.7 ± 15.7	40.8–126.3
BMI (kg/m^2^)	33.6 ± 5.9	20.5–53.1
Affected side		
Left	61 (47.3%)	
Right	68 (52.7%)	
Charlson Comorbidity Index		
Severe	5 (3.9%)	
Mild	16 (12.4%)	
No comorbidities	108 (83.7%)	
Charnley classification [22]		
A	93 (72.1%)	
B1	34 (26.3%)	
C1	1 (0.8%)	
C3	1 (0.8%)	

**Table 3:** Gait scores obtained by the BTS G-Walk gait analysis system.

	Mean ± SD	Range
Speed (m/s)	0.95 ± 0.19	0.58–1.51
Cadence (steps/min)	105.6 ± 9.9	82.3–134
Right stride length (m)	1.25 ± 0.17	0.68–1.64
Left stride length (m)	1.25 ± 0.17	0.69–1.64
Right propulsion (m/s^2^)	5.34 ± 1.98	1.9–12.9
Left propulsion (m/s^2^)	5.43 ± 1.90	2–11.3

**Table 4:** Functional and QoL questionnaires results.

Questionnaire	Mean ± SD	Range
KSS		
Objective	52.81 ± 12.67	20–88
Functional	54.12 ± 21.31	5–90
EQ-5D		
Overall	0.44 ± 0.24	−0.46–0.83
VAS	57.85 ± 18.55	5–95
KOOS		
Symptoms	48.25 ± 20.48	0–96
Pain	37.51 ± 19.69	0–89
ADLs	33.80 ± 19.64	0–76.47
Sport/recreation function	5.32 ± 13.34	0–100
QoL	24.07 ± 17.31	0–81
Total	29.77 ± 13.99	3–69

**Table 5:** Correlation between the scores on the different gait parameters and the results of the KOOS, KSS, EQ-5D, and VAS questionnaires. All results were statistically significant (*p* < 0.05).

Gait Parameters	KSS		KOOS						EQ-5D	
	Objective	Functional	Symptoms	Pain	ADLs	Sport/Recreation Function	QoL	Overall Score	Overall	VAS
Speed	0.183	0.161	0.257	0.271	0.476 *	0.148	0.240	0.371 *	0.245	0.223
Cadence	0.007	0.193	0.025	0.060	0.230	0.113	0.112	0.140	0.039	0.011
Right stride length	0.188	0.084	0.264	0.280	0.412 *	0.119	0.212	0.343 *	0.232	0.250
Left stride length	0.194	0.085	0.262	0.276	0.410 *	0.120	0.215	0.342 *	0.234	0.247
Right propulsion	0.192	0.145	0.233	0.226	0.445 *	0.147	0.159	0.319 *	0.207	0.191
Left propulsion	0.085	0.083	0.131	0.143	0.382 *	0.163	0.130	0.246	0.218	0.175

* Indicates that the value is associated with a low correlation. All other values are indicative of a lack of correlation.

## Data Availability

The data presented in this study are available on request from the corresponding author.

## Supplementary Materials

The following supporting information can be downloaded at: https://www.mdpi.com/article/10.3390/jfmk8020077/s1, Functional evaluation questionnaire S1: Knee Society Score (KSS) questionnaire, Generic QoL evaluation questionnaire S2: EQ-5D-5L questionnaire, Knee osteoarthritis-specific QoL evaluation questionnaire S3: Knee Injury and Osteoarthritis Outcome Score (KOOS).

## References

[1] Felson D.T. (2000). Osteoarthritis: New Insights. Part 1: The Disease and Its Risk Factors. Ann. Intern. Med..

[2] Kingsbury S.R., Gross H.J., Isherwood G., Conaghan P.G. (2014). Osteoarthritis in Europe: Impact on Health Status, Work Productivity and Use of Pharmacotherapies in Five European Countries. Rheumatology.

[3] The Whoqol Group (1998). Development of the World Health Organization WHOQOL-BREF Quality of Life Assessment. Psychol. Med..

[4] Kawano M.M., Araújo I.L.A., Castro M.C., Matos M.A. (2015). Assessment of Quality of Life in Patients with Knee Osteoarthritis. Acta Ortopédica Bras..

[5] Farr II J., Miller L.E., Block J.E. (2013). Quality of Life in Patients with Knee Osteoarthritis: A Commentary on Nonsurgical and Surgical Treatments. Open Orthop. J..

[6] Bernad-Pineda M., de las Heras-Sotos J., Garcés-Puentes M.V. (2014). Quality of Life in Patients with Knee and Hip Osteoarthritis. Rev. Española Cirugía Ortopédica Traumatol..

[7] Neuprez A., Neuprez A.H., Kurth W., Gillet P., Bruyère O., Reginster J.-Y. (2018). Profile of Osteoarthritic Patients Undergoing Hip or Knee Arthroplasty, a Step toward a Definition of the “Need for Surgery”. Aging Clin. Exp. Res..

[8] Elbadawy M.A. (2017). Effectiveness of Periosteal Stimulation Therapy and Home Exercise Program in the Rehabilitation of Patients With Advanced Knee Osteoarthritis. Clin. J. Pain.

[9] Akimoto T., Kawamura K., Wada T., Ishihara N., Yokota A., Suginoshita T., Yokoyama S. (2022). Evaluation of Gait Cycle Time Variability in Patients with Knee Osteoarthritis Using a Triaxial Accelerometer. medRxiv.

[10] Bączkowicz D., Skiba G., Czerner M., Majorczyk E. (2018). Gait and Functional Status Analysis before and after Total Knee Arthroplasty. Knee.

[11] Pirker W., Katzenschlager R. (2017). Gait Disorders in Adults and the Elderly. Wien. Klin. Wochenschr..

[12] Favre J., Jolles B.M. (2016). Gait Analysis of Patients with Knee Osteoarthritis Highlights a Pathological Mechanical Pathway and Provides a Basis for Therapeutic Interventions. EFORT Open Rev..

[13] Mancuso C.A., Sculco T.P., Wickiewicz T.L., Jones E.C., Robbins L., Warren R.F., Williams-Russo P. (2001). Patients’ Expectations of Knee Surgery. J. Bone Jt. Surg. -Am. Vol..

[14] Yoo J.H., Chang C.B., Kang Y.G., Kim S.J., Seong S.C., Kim T.K. (2011). Patient Expectations of Total Knee Replacement and Their Association with Sociodemographic Factors and Functional Status. J. Bone Jt. Surg. Br..

[15] Ismailidis P., Hegglin L., Egloff C., Pagenstert G., Kernen R., Eckardt A., Ilchmann T., Nüesch C., Mündermann A. (2021). Side to Side Kinematic Gait Differences within Patients and Spatiotemporal and Kinematic Gait Differences between Patients with Severe Knee Osteoarthritis and Controls Measured with Inertial Sensors. Gait Posture.

[16] van Uden C.J., Besser M.P. (2004). Test-Retest Reliability of Temporal and Spatial Gait Characteristics Measured with an Instrumented Walkway System (GAITRite^®^). BMC Musculoskelet. Disord..

[17] Brandes M., Schomaker R., Möllenhoff G., Rosenbaum D. (2008). Quantity versus Quality of Gait and Quality of Life in Patients with Osteoarthritis. Gait Posture.

[18] Pequeno N.P.F., Cabral N.L.d.A., Marchioni D.M., Lima S.C.V.C., Lyra C. (2020). de O. Quality of Life Assessment Instruments for Adults: A Systematic Review of Population-Based Studies. Health Qual. Life Outcomes.

[19] Debi R., Mor A., Segal G., Segal O., Agar G., Debbi E., Halperin N., Haim A., Elbaz A. (2011). Correlation between Single Limb Support Phase and Self-Evaluation Questionnaires in Knee Osteoarthritis Populations. Disabil. Rehabil..

[20] Hyun C.W., Kim B.R., Han E.Y., Kim S.R. (2016). Preoperative Physical Performance Predictors of Self-Reported Physical Function and Quality of Life in Patients Scheduled for Total Knee Arthroplasty. J. Phys. Ther. Sci..

[21] Kohn M.D., Sassoon A.A., Fernando N.D. (2016). Classifications in Brief: Kellgren-Lawrence Classification of Osteoarthritis. Clin. Orthop. Relat. Res..

[22] Harris A.I., Luo T.D., Lang J.E., Kopjar B. (2018). Short-Term Safety and Effectiveness of a Second-Generation Motion-Guided Total Knee System. Arthroplast. Today.

[23] Gieysztor E., Pecuch A., Kowal M., Borowicz W., Paprocka-Borowicz M. (2020). Pelvic Symmetry Is Influenced by Asymmetrical Tonic Neck Reflex during Young Children’s Gait. Int. J. Environ. Res. Public Health.

[24] INSALL J.N., DORR L.D., SCOTT R.D., NORMAN W. (1989). Rationale, of The Knee Society Clinical Rating System. Clin. Orthop. Relat. Res..

[25] Hernandez G., Garin O., Pardo Y., Vilagut G., Pont À., Suárez M., Neira M., Rajmil L., Gorostiza I., Ramallo-Fariña Y. (2018). Validity of the EQ–5D–5L and Reference Norms for the Spanish Population. Qual. Life Res..

[26] Vaquero J., Longo U.G., Forriol F., Martinelli N., Vethencourt R., Denaro V. (2014). Reliability, Validity and Responsiveness of the Spanish Version of the Knee Injury and Osteoarthritis Outcome Score (KOOS) in Patients with Chondral Lesion of the Knee. Knee Surg. Sport. Traumatol. Arthrosc..

[27] Hinkle D.E., Wiersma W., Jurs S.G. (2003). Applied Statistics for the Behavioral Sciences.

[28] Abellan Van Kan G., Rolland Y., Andrieu S., Bauer J., Beauchet O., Bonnefoy M., Cesari M., Donini L.M., Gillette-Guyonnet S., Inzitari M. (2009). Gait Speed at Usual Pace as a Predictor of Adverse Outcomes in Community-Dwelling Older People an International Academy on Nutrition and Aging (IANA) Task Force. J. Nutr. Health Aging.

[29] Moe-Nilssen R., Helbostad J.L. (2020). Spatiotemporal Gait Parameters for Older Adults—An Interactive Model Adjusting Reference Data for Gender, Age, and Body Height. Gait Posture.

[30] Jerome G.J., Ko S., Kauffman D., Studenski S.A., Ferrucci L., Simonsick E.M. (2015). Gait Characteristics Associated with Walking Speed Decline in Older Adults: Results from the Baltimore Longitudinal Study of Aging. Arch. Gerontol. Geriatr..

[31] Oatis C.A., Wolff E.F., Lockard M.A., Michener L.A., Robbins S.J. (2013). Correlations among Measures of Knee Stiffness, Gait Performance and Complaints in Individuals with Knee Osteoarthritis. Clin. Biomech..

[32] Fransen B.L., Pijnappels M., Butter I.K., Burger B.J., van Dieën J.H., Hoozemans M.J.M. (2022). Patients’ Perceived Walking Abilities, Daily-Life Gait Behavior and Gait Quality before and 3 Months after Total Knee Arthroplasty. Arch. Orthop. Trauma Surg..

[33] Elkarif V., Kandel L., Rand D., Schwartz I., Greenberg A., Portnoy S. (2020). Kinematics Following Gait Perturbation in Adults with Knee Osteoarthritis: Scheduled versus Not Scheduled for Knee Arthroplasty. Gait Posture.

[34] Baert I.A.C., Jonkers I., Staes F., Luyten F.P., Truijen S., Verschueren S.M.P. (2013). Gait Characteristics and Lower Limb Muscle Strength in Women with Early and Established Knee Osteoarthritis. Clin. Biomech..

[35] Miralles-Muñoz F.A., Gonzalez-Parreño S., Martinez-Mendez D., Gonzalez-Navarro B., Ruiz-Lozano M., Lizaur-Utrilla A., Alonso-Montero C. (2022). A Validated Outcome Categorization of the Knee Society Score for Total Knee Arthroplasty. Knee Surg. Sport. Traumatol. Arthrosc..

[36] Garcia-Gordillo M.A., Adsuar J.C., Olivares P.R. (2016). Normative Values of EQ-5D-5L: In a Spanish Representative Population Sample from Spanish Health Survey, 2011. Qual. Life Res..

[37] Paradowski P.T., Bergman S., Sundén-Lundius A., Lohmander L.S., Roos E.M. (2006). Knee Complaints Vary with Age and Gender in the Adult Population. Population-Based Reference Data for the Knee Injury and Osteoarthritis Outcome Score (KOOS). BMC Musculoskelet. Disord..

[38] Chang J., Fu M., Cao P., Ding C., Wang D. (2022). Patient-Reported Quality of Life Before and After Total Knee Arthroplasty: A Multicenter Observational Study. Patient Prefer. Adherence.

[39] Xie F., Li S.-C., Roos E.M., Fong K.-Y., Lo N.-N., Yeo S.-J., Yang K.-Y., Yeo W., Chong H.-C., Thumboo J. (2006). Cross-Cultural Adaptation and Validation of Singapore English and Chinese Versions of the Knee Injury and Osteoarthritis Outcome Score (KOOS) in Asians with Knee Osteoarthritis in Singapore. Osteoarthr. Cartil..

[40] Wojcieszek A., Kurowska A., Majda A., Liszka H., Gądek A. (2022). The Impact of Chronic Pain, Stiffness and Difficulties in Performing Daily Activities on the Quality of Life of Older Patients with Knee Osteoarthritis. Int. J. Environ. Res. Public Health.

[41] Debi R., Mor A., Elbaz A., Segal G., Lubovsky O., Kahn G., Peskin B., Beer Y., Atoun E. (2017). Correlation between Gait Analysis and Clinical Questionnaires in Patients with Spontaneous Osteonecrosis of the Knee. Clin. Biomech..

[42] Turcot K., Sagawa Y., Fritschy D., Hoffmeyer P., Suvà D., Armand S. (2013). How Gait and Clinical Outcomes Contribute to Patients’ Satisfaction Three Months Following A Total Knee Arthroplasty. J. Arthroplast..

[43] Yamashina S., Harada K., Tanaka R., Inoue Y. (2023). Abnormal Gait Pattern Examination Screening for Physical Activity Level after One Year in Patients with Knee Osteoarthritis. J. Funct. Morphol. Kinesiol..

[44] Sparkes V., Whatling G.M., Biggs P., Khatib N., Al-Amri M., Williams D., Hemming R., Hagen M., Saleem I., Swaminathan R. (2019). Comparison of Gait, Functional Activities, and Patient-Reported Outcome Measures in Patients with Knee Osteoarthritis and Healthy Adults Using 3D Motion Analysis and Activity Monitoring: An Exploratory Case-Control Analysis. Orthop. Res. Rev..

[45] Guiotto A., Bortolami G., Ciniglio A., Spolaor F., Guarneri G., Avogaro A., Cibin F., Silvestri F., Sawacha Z. (2022). Machine Learning Approach to Diabetic Foot Risk Classification with Biomechanics Data. Gait Posture.

[46] Song Y., Cen X., Chen H., Sun D., Munivrana G., Bálint K., Bíró I., Gu Y. (2023). The Influence of Running Shoe with Different Carbon-Fiber Plate Designs on Internal Foot Mechanics: A Pilot Computational Analysis. J. Biomech..

[47] Pan D., TianYe L., Peng Y., JingLi X., HongZhu L., HeRan Z., QingWen Z., LeiLei C., ZhenQiu C., QiuShi W. (2020). Effects of Proximal Fibular Osteotomy on Stress Changes in Mild Knee Osteoarthritis with Varus Deformity: A Finite Element Analysis. J. Orthop. Surg. Res..

