# Supplementary material for: Low Correlation between Gait and Quality of Life in Advanced Knee Osteoarthritis
Source: J Funct Morphol Kinesiol. 2023 Jun 8;8(2):77. doi: 10.3390/jfmk8020077 (PMC10299091; doi:10.3390/jfmk8020077)
Supplement: Supplementary file 1 [file jfmk-08-00077-s001.zip › jfmk-2415667-supplementary.pdf]

Functional evaluation questionnaire S1: Knee Society Score (KSS) questionnaire

KNEE SOCIETY SCORE: PRE-OP

DEMOGRAPHIC INFORMATION (To be completed by patient)

1- Today's date

☐☐ / ☐☐ / ☐☐☐☐

Enter dates as: mm/dd/yyyy

2- Date of birth

☐☐ / ☐☐ / ☐☐☐☐

3- Height (ft' in")

☐ ☐☐

4- Weight (lbs.)

☐☐☐

5- Sex

○ Male ○ Female

6- Side of this (symptomatic) knee

○ Left ○ Right

If both knees will be operated on, please use a different form for each knee

7- Ethnicity

○ Native Hawaiian or other Pacific Islander ○ American Indian or Alaska Native ○ Hispanic or Latino

○ Arab or Middle Eastern ○ African American or Black ○ Asian ○ White

8- Please indicate the expected date and surgeon for your knee replacement operation

Date

☐☐ / ☐☐ / ☐☐☐☐

Enter dates as: mm/dd/yyyy

Name of Surgeon

☐

9- Will this be a primary or revision knee replacement?

○ Primary ○ Revision

To be completed by surgeon

10- Charnley Functional Classification (Use Code Below) ☐

A	Unilateral Knee Arthritis	C1	TKR, but remote arthritis affecting ambulation
B1	Unilateral TKA, opposite knee arthritic	C2	TKR, but medical condition affecting ambulation
B2	Bilateral TKA	C3	Unilateral or Bilateral TKA with Unilateral or Bilateral THR

© 2011 by The Knee Society. All rights reserved. No part of this document may be reproduced or transmitted in any form or by any means, electronic, mechanical, photocopying, recording, or otherwise, without prior written permission of The Knee Society.

OBJECTIVE KNEE INDICATORS (To be completed by surgeon)

ALIGNMENT

1- Alignment: measured on AP standing Xray (Anatomic Alignment) **25 point max**

Neutral: 2-10 degrees valgus (25 pts)
Varus: < 2 degrees valgus (-10 pts)
Valgus: > 10 degrees valgus (-10 pts)

INSTABILITY

2- Medial / Lateral Instability: measured in full extension **15 point max**

None (15 pts)
Little or < 5 mm (10 pts)
Moderate or 5 mm (5 pts)
Severe or > 5 mm (0 pts)

3- Anterior / Posterior Instability: measured at 90 degrees **10 point max**

None (10 pts)
Moderate < 5 mm (5 pts)
Severe > 5 mm (0 pts)

JOINT MOTION

4- Range of motion (1 point for each 5 degrees)

Deductions

Flexion Contracture **Minus Points**
1-5 degrees (-2 pts)
6-10 degrees (-5 pts)
11-15 degrees (-10 pts)
> 15 degrees (-15 pts)

Extensor Lag **Minus Points**
<10 degrees (-5 pts)
10-20 degrees (-10 pts)
> 20 degrees (-15 pts)

© 2011 by The Knee Society. All rights reserved. No part of this document may be reproduced or transmitted in any form or by any means, electronic, mechanical, photocopying, recording, or otherwise, without prior written permission of The Knee Society.

SYMPTOMS

(To be completed by patient)

1- Pain with level walking (10 - Score)

0	1	2	3	4	5	6	7	8	9	10

none severe

2- Pain with stairs or inclines (10 - Score)

0	1	2	3	4	5	6	7	8	9	10

none severe

3- Does this knee feel "normal" to you? (5 points)

- ○ Always (5 pts)
- ○ Sometimes (3 pts)
- ○ Never (0 pts)

Maximum total points (25 points)

PATIENT SATISFACTION

1- Currently, how satisfied are you with the pain level of your knee while sitting? (8 points)

○ Very Satisfied (8 pts) ○ Satisfied (6 pts) ○ Neutral (4 pts) ○ Dissatisfied (2 pts) ○ Very Dissatisfied (0 pts)

2- Currently, how satisfied are you with the pain level of your knee while lying in bed? (8 points)

○ Very Satisfied (8 pts) ○ Satisfied (6 pts) ○ Neutral (4 pts) ○ Dissatisfied (2 pts) ○ Very Dissatisfied (0 pts)

3- Currently, how satisfied are you with your knee function while getting out of bed? (8 points)

○ Very Satisfied (8 pts) ○ Satisfied (6 pts) ○ Neutral (4 pts) ○ Dissatisfied (2 pts) ○ Very Dissatisfied (0 pts)

4- Currently, how satisfied are you with your knee function while performing light household duties? (8 points)

○ Very Satisfied (8 pts) ○ Satisfied (6 pts) ○ Neutral (4 pts) ○ Dissatisfied (2 pts) ○ Very Dissatisfied (0 pts)

5- Currently, how satisfied are you with your knee function while performing leisure recreational activities? (8 points)

○ Very Satisfied (8 pts) ○ Satisfied (6 pts) ○ Neutral (4 pts) ○ Dissatisfied (2 pts) ○ Very Dissatisfied (0 pts)

Maximum total points (40 points)

© 2011 by The Knee Society. All rights reserved. No part of this document may be reproduced or transmitted in any form or by any means, electronic, mechanical, photocopying, recording, or otherwise, without prior written permission of The Knee Society.

PATIENT EXPECTATIONS (To be completed by patient)

What do you expect to accomplish with your knee replacement:

1- Do you expect your knee joint replacement surgery will relieve your knee pain? **(5 points)**

- ○ no, not at all (1 pt)
- ○ yes, a little bit (2 pts)
- ○ yes, somewhat (3 pts)
- ○ yes, a moderate amount (4 pts)
- ○ yes, a lot (5 pts)

2- Do you expect your surgery will help you carry out your normal activities of daily living? **(5 points)**

- ○ no, not at all (1 pt)
- ○ yes, a little bit (2 pts)
- ○ yes, somewhat (3 pts)
- ○ yes, a moderate amount (4 pts)
- ○ yes, a lot (5 pts)

3- Do you expect you surgery will help you perform leisure, recreational or sports activities? **(5 points)**

- ○ no, not at all (1 pt)
- ○ yes, a little bit (2 pts)
- ○ yes, somewhat (3 pts)
- ○ yes, a moderate amount (4 pts)
- ○ yes, a lot (5 pts)

Maximum total points (15 points) ☐

© 2011 by The Knee Society. All rights reserved. No part of this document may be reproduced or transmitted in any form or by any means, electronic, mechanical, photocopying, recording, or otherwise, without prior written permission of The Knee Society.

FUNCTIONAL ACTIVITIES (To be completed by patient)

WALKING AND STANDING (30 points)

1 - Can you walk without any aids (such as a cane, crutches or wheelchair)? **(0 points)**

○ Yes ○ No

2 - If no, which of the following aid(s) do you use? **(-10 points)**

○ wheelchair (-10 pts) ○ walker (-8 pts) ○ crutches (-8 pts) ○ two canes (-6 pts) ☐

○ one crutch (-4 pts) ○ one cane (-4 pts) ○ knee sleeve / brace (-2 pts)

○ other ☐☐☐☐☐☐☐☐☐☐☐☐☐☐☐☐☐☐☐☐

3 - Do you use these aid(s) because of your knees? **(0 points)**

○ Yes ○ No

4 - For how long can you stand (with or without aid) before sitting due to knee discomfort? **(15 points)**

○ cannot stand (0 pts) ○ 0-5 minutes (3 pts) ○ 6-15 minutes (6 pts) ☐

○ 16-30 minutes (9 pts) ○ 31-60 minutes (12 pts) ○ more than an hour (15 pts)

5 - For how long can you walk (with or without aid) before stopping due to knee discomfort? **(15 points)**

○ cannot walk (0 pts) ○ 0-5 minutes (3 pts) ○ 6-15 minutes (6 pts) ☐

○ 16-30 minutes (9 pts) ○ 31-60 minutes (12 pts) ○ more than an hour (15 pts)

Maximum points (30 points) ☐

© 2011 by The Knee Society. All rights reserved. No part of this document may be reproduced or transmitted in any form or by any means, electronic, mechanical, photocopying, recording, or otherwise, without prior written permission of The Knee Society.

STANDARD ACTIVITIES (30 points)

How much does your knee bother you during each of the following activities?	no bother 5	slight 4	moderate 3	severe 2	very severe 1	cannot do (because of knee) 0	I never do this	
1 - Walking on an uneven surface	○	○	○	○	○	○	○	
2 - Turning or pivoting on your leg	○	○	○	○	○	○	○	
3 - Climbing up or down a flight of stairs	○	○	○	○	○	○	○	
4 - Getting up from a low couch or a chair without arms	○	○	○	○	○	○	○	
5 - Getting into or out of a car	○	○	○	○	○	○	○	
6 - Moving laterally (stepping to the side)	○	○	○	○	○	○	○	

Maximum points (30 points)

ADVANCED ACTIVITIES (25 points)

1 - Climbing a ladder or step stool	○	○	○	○	○	○	○	
2 - Carrying a shopping bag for a block	○	○	○	○	○	○	○	
3 - Squatting	○	○	○	○	○	○	○	
4 - Kneeling	○	○	○	○	○	○	○	
5 - Running	○	○	○	○	○	○	○	

Maximum points (25 points)

© 2011 by The Knee Society. All rights reserved. No part of this document may be reproduced or transmitted in any form or by any means, electronic, mechanical, photocopying, recording, or otherwise, without prior written permission of The Knee Society.

DISCRETIONARY KNEE ACTIVITIES (15 points)

Please check 3 of the activities below that you consider *most important* to you.

(Please do not write in additional activities)

Recreational Activities

- ☐ Swimming
- ☐ Golfing (18 holes)
- ☐ Road Cycling (>30mins)
- ☐ Gardening
- ☐ Bowling
- ☐ Racquet Sports (Tennis, Racquetball, etc.)
- ☐ Distance Walking
- ☐ Dancing / Ballet
- ☐ Stretching Exercises (stretching out your muscles)

Workout and Gym Activities

- ☐ Weight-lifting
- ☐ Leg Extensions
- ☐ Stair-Climber
- ☐ Stationary Biking / Spinning
- ☐ Leg Press
- ☐ Jogging
- ☐ Elliptical Trainer
- ☐ Aerobic Exercises

Please copy all 3 checked activities into the empty boxes below.

How much does your knee bother you during each of these activities?

Activity (Please write the 3 activites from list above)	no bother 5	slight 4	moderate 3	severe 2	very severe 1	cannot do (because of knee) 0	
1.	○	○	○	○	○	○	☐
2.	○	○	○	○	○	○	☐
3.	○	○	○	○	○	○	☐

Maximum points (15 points) ☐

Maximum total points (100 points) ☐

© 2011 by The Knee Society. All rights reserved. No part of this document may be reproduced or transmitted in any form or by any means, electronic, mechanical, photocopying, recording, or otherwise, without prior written permission of The Knee Society.

Generic QoL evaluation questionnaire S2: EQ-5D-5L questionnaire

EQ-5D-5L

Sample

Health Questionnaire

English version for the UK

© 2009 EuroQol Research Foundation. EQ-5D™ is a trade mark of the EuroQol Research Foundation. UK (English) v1.2

Under each heading, please tick the ONE box that best describes your health TODAY.

MOBILITY

I have no problems in walking about ❑

I have slight problems in walking about ❑

I have moderate problems in walking about ❑

I have severe problems in walking about ❑

I am unable to walk about ❑

SELF-CARE

I have no problems washing or dressing myself ❑

I have slight problems washing or dressing myself ❑

I have moderate problems washing or dressing myself ❑

I have severe problems washing or dressing myself ❑

I am unable to wash or dress myself ❑

USUAL ACTIVITIES *(e.g. work, study, housework, family or leisure activities)*

I have no problems doing my usual activities ❑

I have slight problems doing my usual activities ❑

I have moderate problems doing my usual activities ❑

I have severe problems doing my usual activities ❑

I am unable to do my usual activities ❑

PAIN / DISCOMFORT

I have no pain or discomfort ❑

I have slight pain or discomfort ❑

I have moderate pain or discomfort ❑

I have severe pain or discomfort ❑

I have extreme pain or discomfort ❑

ANXIETY / DEPRESSION

I am not anxious or depressed ❑

I am slightly anxious or depressed ❑

I am moderately anxious or depressed ❑

I am severely anxious or depressed ❑

I am extremely anxious or depressed ❑

Sample

© 2009 EuroQol Research Foundation. EQ-5D™ is a trade mark of the EuroQol Research Foundation. UK (English) v1.2

- We would like to know how good or bad your health is TODAY.
- This scale is numbered from 0 to 100.
- 100 means the best health you can imagine.
  0 means the worst health you can imagine.
- Please mark an X on the scale to indicate how your health is TODAY.
- Now, write the number you marked on the scale in the box below.

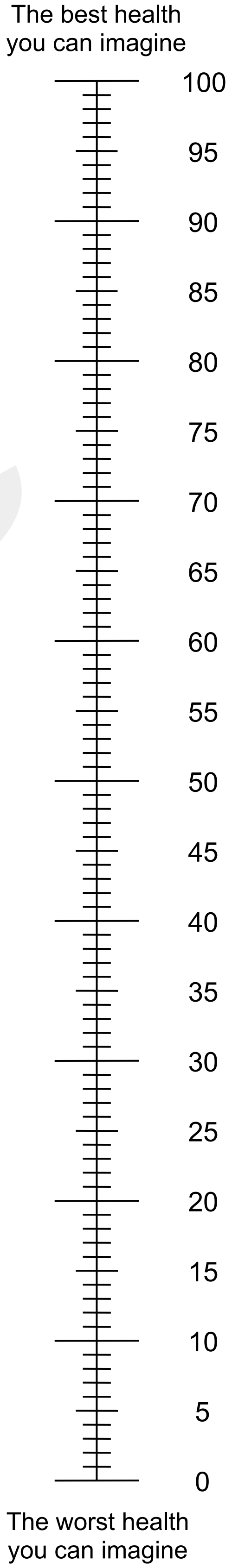

YOUR HEALTH TODAY = ☐

Sample

© 2009 EuroQol Research Foundation. EQ-5D™ is a trade mark of the EuroQol Research Foundation. UK (English) v1.2

Knee osteoarthritis-specific QoL evaluation questionnaire S3: Knee Injury and Osteoarthritis Outcome Score (KOOS)

Knee Injury and Osteoarthritis Outcome Score (KOOS)

Source: Roos EM, Roos HP, Lohmander LS, Ekdahl C, Beynnon BD. Knee Injury and Osteoarthritis Outcome Score (KOOS)--development of a self-administered outcome measure. *J Orthop Sports Phys Ther*. 1998 Aug;28(2):88-96.

The Knee Injury and Osteoarthritis Outcome Score (KOOS) is a questionnaire designed to assess short and long-term patient-relevant outcomes following knee injury. The KOOS is self-administered and assesses five outcomes: pain, symptoms, activities of daily living, sport and recreation function, and knee-related quality of life. The KOOS meets basic criteria of outcome measures and can be used to evaluate the course of knee injury and treatment outcome. KOOS is patient-administered, the format is user-friendly and it takes about 10 minutes to fill out.

Scoring instructions

The KOOS's five patient-relevant dimensions are scored separately: Pain (nine items); Symptoms (seven items); ADL Function (17 items); Sport and Recreation Function (five items); Quality of Life (four items). A Likert scale is used and all items have five possible answer options scored from 0 (No problems) to 4 (Extreme problems) and each of the five scores is calculated as the sum of the items included.

Interpretation of scores

Scores are transformed to a 0–100 scale, with zero representing extreme knee problems and 100 representing no knee problems as common in orthopaedic scales and generic measures. Scores between 0 and 100 represent the percentage of total possible score achieved.

Knee Injury and Osteoarthritis Outcome Score (KOOS)

Pain

P1 How often is your knee painful?	☐ Never	☐ Monthly	☐ Weekly	☐ Daily	☐ Always

What degree of pain have you experienced the last week when…?

P2 Twisting/pivoting on your knee	☐ None	☐ Mild	☐ Moderate	☐ Severe	☐ Extreme
P3 Straightening knee fully	☐ None	☐ Mild	☐ Moderate	☐ Severe	☐ Extreme
P4 Bending knee fully	☐ None	☐ Mild	☐ Moderate	☐ Severe	☐ Extreme
P5 Walking on flat surface	☐ None	☐ Mild	☐ Moderate	☐ Severe	☐ Extreme
P6 Going up or down stairs	☐ None	☐ Mild	☐ Moderate	☐ Severe	☐ Extreme
P7 At night while in bed	☐ None	☐ Mild	☐ Moderate	☐ Severe	☐ Extreme
P8 Sitting or lying	☐ None	☐ Mild	☐ Moderate	☐ Severe	☐ Extreme
P9 Standing upright	☐ None	☐ Mild	☐ Moderate	☐ Severe	☐ Extreme

Symptoms

Sy1 How severe is your knee stiffness after first wakening in the morning?	☐ None	☐ Mild	☐ Moderate	☐ Severe	☐ Extreme
Sy2 How severe is your knee stiffness after sitting, lying, or resting later in the day?	☐ None	☐ Mild	☐ Moderate	☐ Severe	☐ Extreme
Sy3 Do you have swelling in your knee?	☐ Never	☐ Rarely	☐ Sometimes	☐ Often	☐ Always
Sy4 Do you feel grinding, hear clicking or any other type of noise when your knee moves?	☐ Never	☐ Rarely	☐ Sometimes	☐ Often	☐ Always
Sy5 Does your knee catch or hang up when moving?	☐ Never	☐ Rarely	☐ Sometimes	☐ Often	☐ Always
Sy6 Can you straighten your knee fully?	☐ Always	☐ Often	☐ Sometimes	☐ Rarely	☐ Never
Sy7 Can you bend your knee fully?	☐ Always	☐ Often	☐ Sometimes	☐ Rarely	☐ Never

Activities of daily living

What difficulty have you experienced the last week…?

A1 Descending	☐ None	☐ Mild	☐ Moderate	☐ Severe	☐ Extreme
A2 Ascending stairs	☐ None	☐ Mild	☐ Moderate	☐ Severe	☐ Extreme
A3 Rising from sitting	☐ None	☐ Mild	☐ Moderate	☐ Severe	☐ Extreme
A4 Standing	☐ None	☐ Mild	☐ Moderate	☐ Severe	☐ Extreme
A5 Bending to floor/picking up an object	☐ None	☐ Mild	☐ Moderate	☐ Severe	☐ Extreme
A6 Walking on flat surface	☐ None	☐ Mild	☐ Moderate	☐ Severe	☐ Extreme
A7 Getting in/out of car	☐ None	☐ Mild	☐ Moderate	☐ Severe	☐ Extreme
A8 Going shopping	☐ None	☐ Mild	☐ Moderate	☐ Severe	☐ Extreme
A9 Putting on socks/stockings	☐ None	☐ Mild	☐ Moderate	☐ Severe	☐ Extreme
A10 Rising from bed	☐ None	☐ Mild	☐ Moderate	☐ Severe	☐ Extreme
A11 Taking off socks/stockings	☐ None	☐ Mild	☐ Moderate	☐ Severe	☐ Extreme
A12 Lying in bed (turning over, maintaining knee position)	☐ None	☐ Mild	☐ Moderate	☐ Severe	☐ Extreme
A13 Getting in/out of bath	☐ None	☐ Mild	☐ Moderate	☐ Severe	☐ Extreme
A14 Sitting	☐ None	☐ Mild	☐ Moderate	☐ Severe	☐ Extreme
A15 Getting on/off toilet	☐ None	☐ Mild	☐ Moderate	☐ Severe	☐ Extreme
A16 Heavy domestic duties (shovelling, scrubbing floors, etc)	☐ None	☐ Mild	☐ Moderate	☐ Severe	☐ Extreme
A17 Light domestic duties (cooking, dusting, etc)	☐ None	☐ Mild	☐ Moderate	☐ Severe	☐ Extreme

Sport and recreation function

What difficulty have you experienced the last week…?

Sp1 Squatting	☐ None	☐ Mild	☐ Moderate	☐ Severe	☐ Extreme
Sp2 Running	☐ None	☐ Mild	☐ Moderate	☐ Severe	☐ Extreme
Sp3 Jumping	☐ None	☐ Mild	☐ Moderate	☐ Severe	☐ Extreme
Sp4 Turning/twisting on your injured knee	☐ None	☐ Mild	☐ Moderate	☐ Severe	☐ Extreme
Sp5 Kneeling	☐ None	☐ Mild	☐ Moderate	☐ Severe	☐ Extreme

Knee-related quality of life

Q1 How often are you aware of your knee problems?	☐ Never	☐ Monthly	☐ Weekly	☐ Daily	☐ Always
Q2 Have you modified your lifestyle to avoid potentially damaging activities to your knee?	☐ Not at all	☐ Mildly	☐ Moderately	☐ Severely	☐ Totally
Q3 How troubled are you with lack of confidence in your knee?	☐ Not at all	☐ Mildly	☐ Moderately	☐ Severely	☐ Totally
Q4 In general, how much difficulty do you have with your knee?	☐ None	☐ Mild	☐ Moderate	☐ Severe	☐ Extreme
